# Cancer cell transmission via the placenta

**DOI:** 10.1093/emph/eoy011

**Published:** 2018-04-14

**Authors:** Mel Greaves, William Hughes

**Affiliations:** 1 Centre for Evolution and Cancer, The Institute of Cancer Research, Brookes Lawley Building, London SM2 5NG, UK; 2 School of Life Sciences, University of Sussex, Brighton BN1 9QG, UK

**Keywords:** haemochorial placenta, placental anastomoses, immune evasion, cancer clonal markers, choriocarcinoma

## Abstract

Cancer cells have a parasitic propensity in the primary host but their capacity to transit between individuals is severely restrained by two factors: a lack of a route for viable cell transfer and immune recognition in allogeneic, secondary recipients. Several examples of transmissible animal cancers are now recognised. In humans, the only natural route for transmission is via the haemochorial placenta which is permissive for cell traffic. There are three special examples of this occurring *in utero*: maternal to foetus, intraplacental twin to twin leukaemias and choriocarcinoma-extra-embryonic cells to mother. We discuss the rare circumstances under which such transmission occurs.

## INTRODUCTION

Parasite: *‘An organism that lives in or on another and benefiting at the expense of its host’* (Oxford Modern English Dictionary).

Cancer cells, we suggest, can be considered a unicellular, parasitic entity. They are not generally recognized as such but, by the above definition, it can be argued that they qualify. Cancer has a clonal origin and evolves via selection of cells with adaptive phenotypes within tissue ecosystems [1, 2]. Sequential acquisition of mutations equips cancer cells to become robust [3] and emancipated from constraints on proliferative expansion. They disseminate in the host via a territorial hijack that compromises normal tissue functions, imparting morbidity. This parasitic propensity can be seen as an evolutionary legacy [4].

But there is more to being a successful parasitic entity than simply exploiting a host. Survival or longevity as a parasitic lineage requires a suite of attributes: immune evasion [5] and exploiting a viable route for transmission between individual hosts—coupled with proliferative immortality and maintenance of genome integrity.

For a cancer clone, the most stringent bottleneck is transmission. The other traits are frequently selected during cancer progression in the primary host or patient. For example, genetically unstable cancers can evade immune attack via immuno-editing or loss of histocompatibility locus antigens (HLA) or neoantigens [6]. The capacity of cancer cells to remove their identity tags is even more evident under the strong selective pressure of targeted immunotherapy [7, 8].

Given appropriate selective pressures, enough cells and a high mutation rate, it is perhaps to be expected that cancer clone evolution should, at least very occasionally, enable between-host transmission? The answer to this question is yes, as it has happened in several animal species. We now have several unambiguous examples of transmissible cancers in animal species (Table 1) (reviewed in [9, 10]). In these examples, cancer cells are transferred between hosts by biting, sex or, possibly (for bivalve molluscs), filter-feeding. These well-researched examples of contagious animal cancers are very instructive in terms of immune escape mechanisms and we may be under-estimating the number of examples that exist. Nevertheless, the eight clear examples we have are clonal and it seems reasonable to conclude that the emergence of an immortal lineage of transmissible cancer cells is an extremely rare event.

**Table 1.** Transmissible cancers in animal species

		Transmission route	Immuno-avoidance	References
1.	CTVT (one clone)	Sexual^a^	Down-regulation of MHC	[82, 83]
2.	Tasmanian DFTD (two clones with sub-clonal divergence)	Biting^a^	Down-regulation of MHC + inbred host	[84–86]
3.	Leukaemia in marine bivalves: soft shell clams mussels cockles (two clones) golden carpet shell clams (clone derived from another clam species)	Unknown, but suggested to be via filter feeding	?	[87, 88]
4.	Transmissible Syrian hamster reticulum cell sarcoma (multiple clones)^b^	?	?	[89, 90]

? Uncertain.

a Unclear if transmission of cancer cells is via direct blood contact or via transfer of other fluids (saliva, seminal fluid).

b These early reports have not been followed up and so the status of this example remains uncertain.

## TRANSMISSIBLE CANCER IN HUMANS

Is there evidence that human cancer cells evolve a transmissible, parasitic status? We know human cancers can avoid proliferative senescence and impede or repair telomere attrition [11]. Proliferative immortality is signalled by the cell line HeLa (and murine leukaemia equivalents) that still thrives many decades after the demise of its donor [12] and appears, like canine transmissible venereal tumour (CTVT) and Tasmanian devil facial tumour disease (DFTD), to be genomically or mutationally, complex but stable [13].

But surely, the absence of an accessible route of viable cell transmission and, in particular, the barrier of immune recognition in an outbred species like contemporary *Homo sapiens* renders transmission of cancer between humans, unlike in CTVT and DFTD, highly unlikely? Two unbreachable barriers?

Several decades ago, cancer cells were deliberately transplanted between human individuals in experiments conducted in the 1950s and 1960s that would now be considered unethical. Chester Southam and colleagues at Memorial Sloan Kettering Cancer Center inoculated cancer cells between cancer patients and from cancer patients into ‘volunteers’ from a State Penitentiary [14]. With one exception [15], no injected tumours grew beyond a nodule stage, presumably because of immune rejection.

In a less fortunate case, melanoma cells from a patient were injected into her 80-year-old mother, in an attempt to elicit anti-tumour immunity. The recipient died with disseminated melanoma some 15 months later, presumed to be originating from the injected cells [16]. Allogenic organ or blood transplantation into immuno-suppressed individuals has inadvertently provided an iatrogenic route for cancer cell transfer between individuals (Table 2).

**Table 2.** Examples of inter-person transfer of cancer

	Transmission route	Immuno-avoidance	References
1.	Iatrogenic		
	Incidental transfer of unsuspected cancer with transplanted organs.	Recipient immuno-suppressed.	[91, 92]
	Donor cell leukaemia in recipients of bone marrow or blood stem cell transplants.	Recipient immuno-suppressed.	[93, 94]
	Deliberate, immuno-therapeutic transfer.	^a^	[14–16]
	Accidental transfer (needlestick) to medical worker.	^b^	[95–97]
2.	Placental transfer		
	Leukaemia, between monozygotic twins *in utero* (with monochorionic placentas).	Genetically identical.	[58]
	Cross placental from mother to foetus.	Deletion of disparate HLA loci.	[33–35]
	Choriocarcinoma: embryonic trophoblast cells to mother.	Modified HLA expression on trophoblast cells.	[70]
		Immune silencing by trophoblasts.	[25]

a Only grew as nodules at site of injection, except for one case of allogeneic cancer transferred to another cancer patient that metastasized [15].

b Includes a transfer from patient to surgeon during an operation [95], accidental inoculation in the hand during biopsy [97] and transfer to a laboratory worker from a cell line [96]. In two of these cases, the transferred cancers grew in recipients as nodules and did not disseminate possibly reflecting immune, HLA disparate, control. However, in one case [97], the cancer metastasized.

Cancer has therefore been transmitted between individuals, albeit, and fortunately, very rarely. And this is under highly contrived circumstances where the two major restraints are breached: a blood route for transmission provided or is naturally available *and* immune recognition is evaded (Table 2). The only natural route available for transfer of cancer cells between individuals is via the placenta.

### Placental anatomy and cell traffic

**Figure 1.**
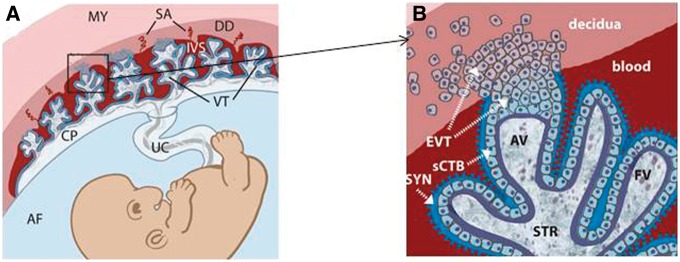
Placental cellular anatomy. Foetus and placenta at 6 weeks gestation. **(A)** MY, myometrium; SA, spiral arteries; DD, decidua; IVS, intervillous space; CP, chorionic plate; UC, umbilical cord; AF, amniotic fluid; VT, villous trophoblast. **(B)** SYN, syncytiotrophoblast; CTB, cytotrophoblast; EVT, extravillous trophoblast; STR, stroma; FV, floating villous; AV, anchoring villous. Taken from Robbins JR, et al; PLoS Pathog 2010; 6(1); e1000732 PubMed 20107601 https://embryology.med.unsw.edu.au/embryology/index.php/Trophoblast

The mammalian placenta is a unique tissue where cells of two genetically different individuals reside in close proximity with direct blood contact [17]. In this context, the developing foetus is effectively an allograft. Humans, in common with other simian primates, have the ancestral type of placental architecture [18] which is both haemochorial and with maternal–foetal villus interdigitation (Fig. 1). This provides an optimised platform for nutritional support in the context of single offspring and long gestational periods [18]. But this anatomical arrangement also brings maternal and foetal cells into a potentially hazardous liaison. Embryonic villous trophoblasts invade and interdigitate into the maternal endometrial decidua and extra-villous trophoblasts remodel maternal arteries, replacing endothelial cells. Embryonic, trophoblast cells are literally bathed in maternal blood.

Effective placental function then requires resolution of two conflicts—inter-genomic resource competition [19, 20] and immunological disparity [21]. Several mechanisms ensure maternal tolerance of paternal antigens on foetal cells within the placenta (reviewed in [22–24]). The solutions employed have clear parallels with immune evasion tactics employed by parasitic species [5]. In the placenta, these include immune-suppression via epigenetic silencing of T-cell attracting cytokine genes in the maternal decidua [25]. Critically also, embryonic trophoblasts at the foetal–maternal interface do not express classical and polymorphic HLA-A and B proteins. In their place are non-polymorphic HLA-G, C and E molecules [26] which may block NK cell and dendritic cell reactivity [24] and promote invasiveness [27]. Additionally, trophoblast cells express PDL1 (programmed death ligand 1), a key negative regulator of immune responses [28]. The trophoblastic interface is immunologically invisible. As it needs to be to avoid rejection of the developing embryo and foetus.

Despite its immunological quiescence, the maternal–foetal interface is not impermeable to cell traffic. It is well documented that normal blood cells migrate between mother and foetus, and vice versa, resulting in micro-chimaerism [29, 30]. It is perhaps then unsurprising that cancer cells can occasionally exploit this opportunity. Transmission of cancer in dogs and Tasmanian devils may be facilitated by wounding, involving blood contact, angiogenesis, cell motility and invasion, features shared with the placenta.

The placenta provides the only setting, to date, for natural transmission of cancer cells between humans, and there are three specific and exceptional examples of this.

### Maternal–foetal transfer of cancer cells

Transplacental transmission of a maternal cancer to the foetus is exceedingly rare. One in 1000 live births involves a mother with cancer [31] but in only a very small number of cases is maternal-foetal transmission recorded. The first such case was in 1866 [32]. Two reviews more than a decade ago reported 15 published cases [33, 34]. Since then, nine more have been published [35–42] or uncovered in historical publications [43]. Given that there are more than 100 million births in a year, worldwide, with possibly 500 000 involving a mother with cancer, just 26 or so cases recorded over many decades represent an exceedingly low risk (∼1 in 5 × 10^−5^).

In all the recorded cases of maternal–foetal transmission, cancer in the infant was of the same type as in the mother. Most of the recorded cases are either melanoma or leukaemia/lymphoma (Table 3). This may reflect the inherent capacity of the cell types involved to migrate, infiltrate and metastasize. Where these were exclusively adult type cancers—melanoma or lung cancer [34, 44], their diagnosis in an infant is all the more striking. Formal, genetic evidence that cancer in the infant cases was of maternal derivation is, in most historical cases, either lacking or based solely on sex chromosomes, i.e. a cancer with an XX karyotype in a male infant. In one case, maternal and infant lymphoma shared the same chromosomal translocation t(X; 1) [45]. Unambiguous evidence for a maternal origin comes from two leukaemia cases in which micro-satellite markers in the infant cancer were of maternal origin [35, 36]. Additionally, in one of these cases, we found that both infant and maternal cancer cells shared the identical, clonotypic *BCR-ABL1* leukaemia fusion gene sequence indicating they were derivative of the same clone [35].

**Table 3.** Materno–foetal transmission of cancer

Cancer type		Age at diagnosis in offspring	Genetic markers of maternal cells	Reference
Leukaemia/lymphoma:				
1.	Lymphosarcoma	birth		[98]
2.	Hodgkin’s disease	3 weeks		[99]
3.	NK cell lymphoma	4 weeks	t(X; 11), XX	[45]
4.	AML	20 months	XX	[50]
5.	ALL	9 months		[100]
6.	ALL	5 months	Karyotype	[101]
7.	B cell lymphoma	8 months	XX	[37]
8.	ALL	11 months	Micro-satellite markers + *BCR-ABL1* sequence	[35]
9.	NK/T lymphoma	8 months	Micro-satellite markers	[36]
Melanoma:				
10.		8 months		[102]
11.		11 days		[103]
12.		7 weeks		[104]
13.		7 months		[105]
14.		2 months		[51]
15.		birth		[40]
16.		birth		[32]
17.		7 months	XX	[106]
18.		6 months		[38]
19.		3 months		[42]
20.	Lung adenocarcinoma	2 weeks	XX	[44]
21.	SCLC	5 months	XX	[107]
22.	SCLC	5 months		[39]
23.	Lung adenocarcinoma	2 months		[108]
24.	Neuro-endocrine cervical ca.	8 months		[41]
25.	Breast ca.	14 months		[43]
26.	Hepatic ca.	birth		[32]

Why should a foetus tolerate a maternal cancer which is, in effect, a foreign allograft? One possibility is that the developing immune system is preferentially tolerized by early exposure [46]. Dizygotic twin cattle are blood cell chimaeras [47] and fail to reject twin skin allografts, an observation that led to the discovery of neonatal, immune tolerance [46]. There is evidence that normal human maternal cells that cross over into the developing foetus may induce stable unresponsiveness to maternal antigens via the activation of tolerogenic regulatory T cells [48].

Another possibility is natural selection of antigenic variants. In a case of maternal–foetal transmission *in utero* of a leukaemia, genetic analysis revealed that the offspring’s maternally derived leukaemic cells had deleted the HLA haplotype that was disparate between mother and offspring [35]. Maternal cancer cells that grew in the infant offspring were therefore likely to be immunologically invisible. The same process of natural immuno-selection or -editing is common in endogenous cancer [6, 49] and is likely to happen when there is strong selective pressure on a genetically unstable or variable target. In another case of transmitted leukaemia, the mother was homozygous at HLA loci so the maternally derived cancer cells in the infant will have been immunologically inert or registered as ‘self’ [50].

In two cases of maternal leukaemia transmission, the clinical presentation in the infant was unusual and very different to that in the mother, the leukaemic cells being confined to a jaw tumour [35] or residing in the testis [36]. This suggests some degree of immunological constraint [35] or, possibly, residence in a privileged (or sanctuary) site [36]. In two cases of maternally transmitted melanoma, the tumour, though lethal in the mother, regressed in the infant indicative of immunological recognition [51]. Collectively, these rare cases suggest that there can be recognition of the maternal tumour by the infant but also that several mechanisms of immune evasion are co-opted by these transmitted cancers.

Since normal blood cells readily migrate transplacentally, why should maternal–foetal transmission of cancer be so infrequent? Leukaemia and melanoma do infiltrate the placenta at a rate that is in considerable excess of maternal cancer arising in the offspring [33, 52]. The proximate explanation may, in part, be that only modest numbers of cells readily cross into the foetal circulation and the probability that this migratory population includes an HLA deletion mutant with propagating or stem cell function may be very low. However, given enough proliferating cancer cells and intense immunological pressure, selection of HLA mutants is very likely. A vivid example of this comes from relapse in acute myeloid leukaemia (AML) in the context of an allo T-cell transplant. Transfer of HLA-mismatched T cells from a donor into a recipient with AML can effectively suppress the leukaemia. But relapse is common and, instructively, these relapses usually show deletion of the mismatched HLA loci, again indicative of selection [53, 54].

### Twin to twin dissemination of leukaemia in utero

**Figure 2.**
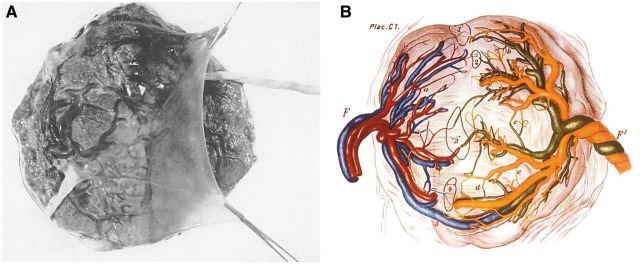
Vascular anatomy of monochorionic, twin placenta. **(A)** Photograph of single, monochorionic placenta with dividing amnion tissue and two umbilical cords. **(B)** Diagrammatic representation of monochorionic twin placenta with vascular anastomoses (labelled 1-5). F and F_1_ indicate umbilical cord of two twins. Taken from Strong and Corney [55]. Original image from Schatz [109]

Dependent upon the timing of splitting of an early embryo, monozygotic twins either share (∼60%) a single, monochorionic placenta (Fig. 2A) or develop in two separate dichorionic placentas [55]. In the 1880s, Schatz described vascular anastomoses in monochorionic placentas (Fig. 2B) [56]. A consequence of this feature is blood cell migration between developing foetuses *in utero* and resultant blood cell chimaerism. Unequal sharing of blood between twins results in the relatively common twin–twin transfusion syndrome in which there is significant morbidity and mortality [57].

A monozygotic pair is no more at risk of any paediatric cancer than a singleton. But a striking feature of cancer in twins is its high rate of concordance of leukaemia. More than 100 cases of concordant acute leukaemia in monozygotic twins have been reported [58], the first being in 1882. Most twin cases of leukaemia are of the common subtype of leukaemia seen in singleton children—B-cell precursor acute lymphoblastic leukaemia (ALL) [59]. The rates of concordance in twin pairs are high, approaching 100% for infants (<18 months) but less, at ∼10–15% for older children. These two have distinctly different, age-associated subtypes of ALL [58].

Early clinical observations on pairs of twins with leukaemia prompted the idea that concordance might arise by leukaemia arising in one twin *in utero*, which then spread to the co-twin via intraplacental anastomoses [60, 61]. The prediction was that paired leukaemias in twins, originating *in utero*, should be monoclonal.

As similar leukaemias in unrelated individuals can harbour the same recurrent chromosomal abnormalities, testing the above hypothesis had to wait until we had robust markers for clonality. This was provided by leukaemia fusion genes [62]. These genetic recombinants are formed following double-strand breaks in the partner genes (usually on separate chromosomes). Breaks occur within a defined intronic breakpoint region but are essentially random or idiosyncratic. The result is that each clone has a unique fusion gene sequence [63]. This then provides stable, sensitive and clone-specific markers.

**Figure 3.**
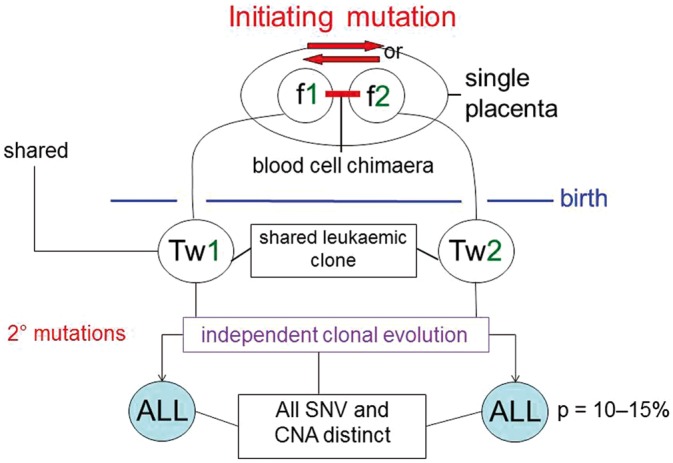
Representation of concordant leukaemia in identical twins. f1, 2, foetus 1, 2; Tw1, 2, twin 1, 2; ALL, acute lymphoblastic leukaemia; SNV, single nucleotide variants; CNA, (gene) copy number alterations; founder lesion, acquired (non-inherited) gene fusion or hyperdiploidy. Diagram illustrates two foetuses in single placenta which share a shared blood circulation. The leukaemia initiating mutation arises in one cell, in one foetus. But the clonal progeny of that cell are then shared by both foetuses and present (and detectable) at birth in both Twins 1 and 2. After birth, essential secondary mutations (CNA and SNV) accumulate independently in the cells of each twin leading to a diagnosis of ALL. If one twin (of a monozygotic, monochorionic pair) has ALL, the probability (p) of the second twin also developing ALL is ∼10–15%, i.e. the secondary genetic changes do not always happen

A systematic genetic analysis in a series of twin pairs using clonal markers revealed that high concordance does indeed derive, not from co-inherited susceptibility genes, but clonally via twin-twin cellular transfer *in utero* [58] (Fig. 3). The sharing of acquired, clone-specific leukaemic mutations indicates that the concordant pairs of leukaemias are monoclonal or arise in one cell, in one twin. The progeny, ‘pre-leukaemic’ cells then disseminate to the co-twin, within the placenta. This only occurs in those monozygotic twins that have a single or monochorionic placenta [58]. Further mutational changes occur after birth, independently in twins, that convert the covert pre-leukaemic clone to overt, clinical leukaemia [64–66]. These secondary genetic events may or (more often) may not arise, hence the concordance for older children is 10–15%, not 100%. In twin pairs *dis*cordant for clinical ALL, the co-twin who remains leukaemia-free nevertheless retains covert pre-leukaemic cells that share the same initiating genetic lesion as in the twin with overt ALL, but are effectively ‘frozen’ in their clonal evolution [65, 67].

The exceptionally high rate of concordance in infant (<18 months) leukaemia [58] suggests the initiating genetic lesion *in utero*—usually an *MLL* fusion gene [62], is sufficient for overt leukaemogenesis and genomic sequencing supports this contention [68, 69]. Transfer of leukaemic or pre-leukaemic cells, when arising *in utero* in one twin, is probably universal when the co-twins are both monozygotic and monochorionic.

There is no evidence that paediatric solid tumour cells, though often of embryonic or foetal origin, spread between twins in this fashion. This may reflect the fact that only leukaemias are blood borne at an early stage in their clonal, pre-natal evolutionary history. Clearly in the monozygotic twin context, there is little or no prospect of immune recognition and rejection of cells derived from a genetically identical individual. Leukaemic cells derived from a co-twin will be immunologically ignored as ‘self’ unless they express leukaemia-associated neoantigens. This has not been explored. The rarity of twins in humans, and the lack of strong heritability of twinning, means that selection for cancers to evolve transmissibility between twins will be weak. Selection could be greater in other animals in which it is common for offspring to develop *in utero* as twins (or larger groups).

### Choriocarcinoma

Gestational choriocarcinoma arises in the placenta very rarely during pregnancies (∼1 in 50 000) that can be either normal in outcome or aborted. Most frequently, choriocarcinoma derives from a complete hydatidiform mole [70]. The latter are usually androgenic—the product of an egg devoid of maternal chromosomes and with paternal chromosomes only [71]. Hydatidiform moles grow, benignly but tumour-like, as disordered chorionic villi composed of trophoblastic cells and convert to invasive choriocarcinoma in the mother at a low frequency (1–2%). An androgenic tumour, such as a choriocarcinoma derived from a hydatidiform mole has a parallel with sexual parasitism in which the female genome is excluded from a fertilized egg [72].

Although choriocarcinoma is generally identified in a patient sometime after a pregnancy, it may occasionally be identified in the placenta itself by ultrasound or other scans and biopsy. These intraplacental choriocarcinoma can result in disseminated disease in the mother, infant or both. It has recently been shown that in the latter scenario, mother and infant may share a common choriocarcinoma clone [73].

The cancer cells in choriocarcinoma derive from villus cytotrophoblast cells that are probably trophoblastic stem cells [74]. Both normal cytotrophoblasts and their malignant counterparts in choriocarcinoma express matrix metalloproteases facilitating invasive and stem cell self-renewal signalling molecules including Nanog, the Wnt pathway and STAT3 [75, 76] observed in other, common cancers. This suggests the normal placental trophoblast cells inherently express, albeit transiently, tumour-associated properties. Choriocarcinoma readily disseminates to the maternal lungs and other organs but is very sensitive to methotrexate or combination chemotherapy and cure rates are high at over 90% [70]. However, this transmitted cancer is intrinsically malignant and lethal in the absence of effective therapy.

A marked similarity between the biology of placental trophoblasts and cancer cells has long been recognised (reviewed in [77]). The normal function of embryonic trophoblastic cells requires that they are invasive of maternal tissue and, in the human placenta, they are in direct contact with maternal blood and indeed can migrate into it and are detectable in the blood of pregnant women [78] and as mentioned above, can escape immune attack in the placental environment by altered HLA expression and release of immuno-suppressive molecules [25, 26]. These same physiological adaptations of invasiveness, immunological disguise and suppression present mutant trophoblasts—choriocarcinoma cells, with a passport to infiltrate and survive in the maternal blood.

Choriocarcinoma provides the only single example we have to date of serial transmission of human cancer: from extra embryonic tissue to mother and, subsequently, to multiple recipients of donor organs transplanted from that mother [79].

## CONCLUSIONS

Cancer cells are mutant cheaters in multicellularity and, as such, they function as endogenous, unicellular parasites. When provided with a route for viable cell transfer, they can relocate, adapt to immunological challenge and disseminate in a new host individual. Serial transmission of cancer and longevity of the parasitic lineage is restricted to a few animal species and is a very rare evolutionary event, or sequence of selective events involving, to date, the emergence of just eight clones.

The only examples we have to date of natural transmission in humans all reflect a liability inherent in the anatomy and function of the placenta. To date, there are no examples of human cancer transmitted by insect bites, human bites or sex.

Transmissibility is a potentially big advantage to a cancer clone. The surprise is not that it occurs, but that it appears to be so very rare. Immune recognition and the paucity of routes for viable cell trafficking are certainly major restraints [80]. But cancer cell virulence may be another. In parasitic species, there is a trade-off between virulence and transmission and, generally, virulence is modulated so that parasites can transmit before host death occurs [81]. When cancers are well advanced in evolutionary trajectory and metastatic, they are more likely to have the robustness and suite of phenotypes required for transmission. This would include a sizeable stem cell fraction. Cells with self-renewal capacity would be essential for transmission and recapitulation of a cancer. On the other hand, advanced cancers are virulent and more likely to be lethal to the patient. The primary host is therefore likely to die before the cancer can transmit. Even if a cancer does transmit successfully to a second host, then it is likely to be metastatic in this host soon after infection and to kill the secondary host before transmission to a third host can take place. It may therefore be that the intrinsic relationship between transmission potential on the one hand, and metastasis and virulence on the other, in most cancers makes their persistent transmission unlikely. The eight cancer clones that have achieved this in animal species all likely owe their transmission success to having weakened this link.

The rare instances of transmission in humans are nevertheless dramatic and distressing. Although they vividly illustrate transmission potential, evolutionary trajectory is abated as transmission is not serial, and aborts with the first recipient. Not a very successful parasite then; and one that poses no public health risk. Malignant cancer cells are perhaps best described as parasites with incipient transmission potential. The anatomy of the human placenta provides a rare opportunity that is, thankfully, only very rarely exploited.
